# Complete Genome Sequence of *Paenibacillus* sp. Strain E222, a Bacterial Symbiont of an *Epichloë* Fungal Endophyte of Ryegrass

**DOI:** 10.1128/MRA.00786-20

**Published:** 2020-10-08

**Authors:** Daniel A. Bastías, Ruy Jauregui, Emma R. Applegate, Eric Altermann, Stuart D. Card, Linda J. Johnson

**Affiliations:** a AgResearch Limited, Grasslands Research Centre, Palmerston North, New Zealand; b Riddet Institute, Massey University, Palmerston North, New Zealand; University of California, Riverside

## Abstract

We report on the whole-genome sequence of *Paenibacillus* sp. strain E222, a bacterium isolated from a fresh culture of Epichloë festucae var. *lolii*, a mutualistic fungal endophyte of perennial ryegrass. The genome has a size of 7.8 Mb and a G+C content of 46% and encodes 6,796 putative protein-coding genes.

## ANNOUNCEMENT

Many bacteria form symbiotic associations with plant-associated fungi and can be located either within the body or cells of the fungus (termed endosymbionts) or on the body surface of the fungus (termed ectosymbionts). Although these types of bacteria are associated mainly with fungi, they can also directly affect plant fitness by modulating plant performance and/or by regulating the performance of their plant-associated fungi (1). Bacterial symbionts of fungi have been isolated from many fungal species that form intimate associations with plants (e.g., references 2 and 3). Here, we report the draft genome sequence of *Paenibacillus* sp. strain E222, a bacterium isolated from the cultured mycelia of Epichloë festucae var. *lolii*. This fungal strain was isolated from a perennial ryegrass plant according to the method published by Latch and Christensen (1985), with the exception that the agar medium did not contain antibiotics (4). *Epichloë festucae* var. *lolii* has coevolved with perennial ryegrass of the family Poaceae, subfamily Pooideae, with which they form long-lived, mutualistic associations (5). The bacterium was isolated by grinding *Epichloë* fungal mycelia with sterilized glass beads immersed in nutrient broth. After grinding and centrifugation, an aliquot of the supernatant was transferred onto nutrient agar and incubated at 28°C for 48 h in the dark. No other microbial growth was observed.

For sequencing purposes, cells of *Paenibacillus* sp. strain E222 were obtained from a single bacterial colony, transferred to 25 ml of Luria-Bertani broth (pH 8), and incubated at 28°C for 48 h at 300 rpm. The genomic bacterial DNA was extracted using the Qiagen blood and cell culture DNA kit (Bio-Strategy Ltd.) following the manufacturer’s instructions. After extraction, the DNA was precipitated with phenol-chloroform (6), and the pellet was purified using the Zymo Clean and Concentrator-25 kit (Ngaio Diagnostics Ltd.). The genomic DNA was sequenced on a Pacific Biosciences (PacBio) Sequel instrument using a library constructed with the SMRTbell express kit and SMRTbell barcoded overhang adapter kit (PacBio, Inc.) as part of a multiplexed experiment. The run produced 160,303 reads with an average length of 9 kb, an *N*_50_ value of 43.5 kb, and a total output of 1.46 Gb, attaining a coverage of 208-fold. The reads were assembled using Canu version 1.6 with default parameters and an estimated genome size of 5 Mb (7). The assembly process produced one single contig of 7.5 Mb. A BUSCO test was run with this genome assembly using the bacterial database odb9, producing a completeness score of 99.3% (147 complete sets, 0 duplicated, 0 missing, 1 fragmented) (8). A CheckM test produced a completeness score of 99.85 and a contamination score of 0.14 (9).

**FIG 1:**
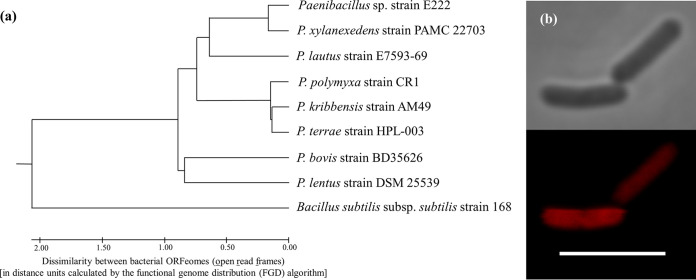
(a) Genome dissimilarity tree between *Paenibacillus* sp. genomes. The genome differences were determined after conducting a functional genome distribution (FGD) analysis (15). This analysis calculates the similitudes between amino acid sequences predicted from bacterial open read frames (ORFeomes). Complete genomes were downloaded from the NCBI genome database and annotated using GAMOLA2 (10) (NCBI accession numbers GCA_001908275.1, GCA_003590055.1, GCA_000507205.2, GCA_002240415.1, GCA_000235585.1, GCA_001421015.2, GCF_003931855.1, and GCA_000009045.1). The tree was inferred using the unweighted pair group method with arithmetic mean (UPGMA) algorithm and drawn to scale in MEGA7 (16), with branch lengths in the same units as those of the distances used to infer the tree. The Bacillus subtilis genome was used as an outgroup. The tree scale bar indicates the dissimilarity between bacterial ORFeomes, and approximated branch lengths are depicted by distance units (dus), which reflect the numeric dissimilarity value calculated by the FGD algorithm (see more details about du calculations in reference 15). (b) Micrographs of *Paenibacillus* sp. E222. Bacterial cells were hybridized with the EUB338 oligonucleotide probe labeled with the Cy3 fluorophore. This probe specifically hybridizes with bacterial 16S rRNA genes (17). The top micrograph shows *Paenibacillus* sp. cells under phase-contrast illumination, whereas the bottom image displays the same cells (and field) hybridized with the EUB338 probe. The micrograph scale bar represents 5 μm.

The whole genome of *Paenibacillus* sp. strain E222 has a size of 7.5 Mb with a G+C content of 46%. The genome annotation was carried out using GAMOLA2 (10). The annotated genome contained 6,932 genes, with 6,796 total coding sequences, 110 tRNA genes, 86 rRNA genes, 113 noncoding RNA genes, and 1 CRISPR/CAS system. The whole *Paenibacillus* sp. E222 genome sequence was most similar to that of Paenibacillus xylanexedens PAMC 22703 (Fig. 1a). *Paenibacillus* sp. E222 has a rod-shaped morphotype like others within this clade and is a facultative symbiont of *E. festucae* var. *lolii* (Fig. 1b). Preliminary microscopic examination of *E. festucae* var. *lolii* mycelia enriched with cells of *Paenibacillus* sp. E222 suggested that this bacterial strain was not an endosymbiont and that it established an ectosymbiotic relationship with this fungal species. In order to predict the potential of *Paenibacillus* sp. E222 to produce secondary metabolites, the genome of this strain was analyzed with the antiSMASH software (version 5.1.0) (11). This analysis predicted that the genome contained nine gene clusters coding for enzymes involved in the biosynthesis of lanthipeptides, type III polyketides, nonribosomal peptides, bacteriocins, lasso peptides, siderophores, and terpenes. Some of these compounds have been identified and isolated from *Paenibacillus* species (12–14).

### Data availability.

The genome assembly and the raw sequence data have been deposited in the NCBI nucleotide and SRA databases under accession numbers CP058552 and PRJNA641937, respectively. Data were also deposited under SRA accession number SRR12094900.

## References

[1] Bastías DA, Johnson LJ, Card SD 2020 Symbiotic bacteria of plant-associated fungi: friends or foes? Curr Opin Plant Biol 56:1–8. doi:10.1016/j.pbi.2019.10.010. 31786411

[2] Frey-Klett P, Garbaye J, Tarkka M 2007 The mycorrhiza helper bacteria revisited. New Phytol 176:22–36. doi:10.1111/j.1469-8137.2007.02191.x. 17803639

[3] Hoffman MT, Arnold AE 2010 Diverse bacteria inhabit living hyphae of phylogenetically diverse fungal endophytes. Appl Environ Microbiol 76:4063–4075. doi:10.1128/AEM.02928-09. 20435775 PMC2893488

[4] Latch GCM, Christensen MJ 1985 Artificial infection of grasses with endophytes. Ann Appl Biol 107:17–24. doi:10.1111/j.1744-7348.1985.tb01543.x.

[5] Schardl CL, Leuchtmann A, Spiering MJ 2004 Symbioses of grasses with seedborne fungal endophytes. Annu Rev Plant Biol 55:315–340. doi:10.1146/annurev.arplant.55.031903.141735. 15377223

[6] Porebski S, Bailey LG, Baum BR 1997 Modification of a CTAB DNA extraction protocol for plants containing high polysaccharide and polyphenol components. Plant Mol Biol Rep 15:8–15. doi:10.1007/BF02772108.

[7] Koren S, Walenz BP, Berlin K, Miller JR, Bergman NH, Phillippy AM 2017 Canu: scalable and accurate long-read assembly via adaptive k-mer weighting and repeat separation. Genome Res 27:722–736. doi:10.1101/gr.215087.116. 28298431 PMC5411767

[8] Simão FA, Waterhouse RM, Ioannidis P, Kriventseva EV, Zdobnov EM 2015 BUSCO: assessing genome assembly and annotation completeness with single-copy orthologs. Bioinformatics 31:3210–3212. doi:10.1093/bioinformatics/btv351. 26059717

[9] Parks DH, Imelfort M, Skennerton CT, Hugenholtz P, Tyson GW 2015 CheckM: assessing the quality of microbial genomes recovered from isolates, single cells, and metagenomes. Genome Res 25:1043–1055. doi:10.1101/gr.186072.114. 25977477 PMC4484387

[10] Altermann E, Lu J, McCulloch A 2017 GAMOLA2, a comprehensive software package for the annotation and curation of draft and complete microbial genomes. Front Microbiol 8:346. doi:10.3389/fmicb.2017.00346. 28386247 PMC5362640

[11] Blin K, Shaw S, Steinke K, Villebro R, Ziemert N, Lee SY, Medema MH, Weber T 2019 antiSMASH 5.0: updates to the secondary metabolite genome mining pipeline. Nucleic Acids Res 47:W81–W87. doi:10.1093/nar/gkz310. 31032519 PMC6602434

[12] Grady EN, MacDonald J, Liu L, Richman A, Yuan Z-C 2016 Current knowledge and perspectives of *Paenibacillus*: a review. Microb Cell Fact 15:203. doi:10.1186/s12934-016-0603-7. 27905924 PMC5134293

[13] Jeong H, Choi S-K, Ryu C-M, Park S-H 2019 Chronicle of a soil bacterium: *Paenibacillus polymyxa* E681 as a tiny guardian of plant and human health. Front Microbiol 10:467. doi:10.3389/fmicb.2019.00467. 30930873 PMC6429003

[14] Baindara P, Nayudu N, Korpole S 2020 Whole genome mining reveals a diverse repertoire of lanthionine synthetases and lanthipeptides among the genus *Paenibacillus*. J Appl Microbiol 128:473–490. doi:10.1111/jam.14495. 31633851

[15] Altermann E 2012 Tracing lifestyle adaptation in prokaryotic genomes. Front Microbiol 3:48. doi:10.3389/fmicb.2012.00048. 22363326 PMC3282942

[16] Kumar S, Stecher G, Tamura K 2016 MEGA7: molecular evolutionary genetics analysis version 7.0 for bigger datasets. Mol Biol Evol 33:1870–1874. doi:10.1093/molbev/msw054. 27004904 PMC8210823

[17] Hugenholtz P, Tyson GW, Blackall LL 2002 Design and evaluation of 16S rRNA-targeted oligonucleotide probes for *fluorescence in situ hybridization*. Methods Mol Biol. 2002;179:29–42. 11692872 10.1385/1-59259-238-4:029

